# Supplementary material for: Carfilzomib’s Real-World Safety Outcomes in Korea: Target Trial Emulation Study Using Electronic Health Records
Source: Int J Environ Res Public Health. 2022 Oct 19;19(20):13560. doi: 10.3390/ijerph192013560 (PMC9603615; doi:10.3390/ijerph192013560)
Supplement: Supplementary file 1 [file ijerph-19-13560-s001.zip › ijerph-1874439-supplementary.pdf]

Carfilzomib's Real-World Safety Outcomes in Korea: Target Trial Emulation Study Using Electronic Health Records

Supplementary Table S1. Target trial protocol of comparative safety analysis of KRd and Rd in relapsed/refractory multiple myeloma.

Supplementary Table S2. Definition of the follow-up duration.

Supplementary Table S3. Definition of the adverse reactions.

Supplementary Table S4. Definition of the covariates.

Supplementary Table S5. Reasons for chemotherapy discontinuation

Supplementary Table S6. Sensitivity analysis by contemporary comparator

Supplementary Figure S1. Forest plot of HR in RWE and OR in ASPIRE trial for each adverse reaction.

Supplementary Table S1. Target trial protocol of comparative safety analysis of KRd and Rd in relapsed/refractory multiple myeloma.

Component	Target Trial	Target Trial Emulation
**Eligibility criteria**	• Adult patients over 18 years-old<br>• Patients with relapsed or refractory multiple myeloma after receiving at least one chemotherapy<br>• Patients receiving KRd or Rd more than once<br>• Patients not participating in clinical trials<br>• Patients with no restrictions on access to electronic medical records	• Same as the target trial
**Treatment strategies**	• Patient group: KRd (carfilzomib 27 mg/m^2 at day 1, 2, 8, 9, 15, 16 + lenalidomide 25 mg at day 1–21 + dexamethasone 20 mg at day1, 8, 15, 22)<br>• Control: Rd (lenalidomide 25 mg at day 1–21 + dexamethasone 20 mg at Day 1, 8, 15, 22)<br>• Allow the use of additional drugs in the treatment.	• Same as the target trial<br>• Allow change of drug administration cycle according to patient condition
**Assignment procedures**	• Randomized, not blinded	• 1:1 propensity score matching
**Follow-up period**	• From the time of treatment assignment, to the first occurrence of each adverse event outcome index or for 30 days after the last drug administration date or up to 19 months.	• Same as the target trial
**Outcome**	• Hematological adverse events (thrombocytopenia)<br>• Non-hematologic adverse reactions	• Same as the target trial

	(dyspnea, hypertension, acute renal failure, heart failure, ischemic heart disease, pulmonary embolism, cough, diarrhea, hypokalemia, fever, upper respiratory tract infection, muscle spasms)	
**Causal contract of interest**	• Per-protocol, intention-to-treat	• Per-protocol
**Statistical analysis plan**	• Continuous variable: mean ± standard deviation, median (range), Student t-test or Mann Whitney U-test<br>• Categorical variables: frequency (%), chi-squared test or Fisher's exact test<br>• Incidence (events/100 person-cycle), hazard ratio (95% confidence interval), Cox regression analysis	• Same as the target trial

KRd, combination regimen of carfilzomib, lenalidomide and dexamethasone; Rd, combination regimen of lenalidomide and dexamethasone

Supplementary Table S2. Definition of the follow-up duration.

Category	Definitions
**Index date**	The date of the first prescription of the treatment within the index period
**Outcome occurrence date**	The date of the first occurrence of each adverse event
**Follow-up end date**	30 days from the date of last drug administration, or up to 19 months, whichever occurs first
**Censoring date**	death, change of treatment regimen, hematopoietic stem cell transplant, follow-up loss, or transfer date, whichever occurs first

Supplementary Table S3. Definition of the adverse reactions.

Adverse Reactions	Definitions
**Dyspnea**	• diagnosis code: R06; no such diagnosis within 60 days from the index date<br>• medical records or nursing record: terms related to dyspnea such as 'out of breath', * **'shortness of breath', 'DOE', 'dyspnea'**
**Hypertension**	• diagnosis code: I10.x, I11.x, I12.x, I13.x, I14.x, I15.x, I16.x; no such diagnosis within 14 days from the index date<br>• medical records: terms related to hypertension such as 'high blood pressure'<br>• drugs: increasing dose of antihypertensive drugs, newly administration of antihypertensive drugs<br>• **†clinical examination: SBP/DBP >140/90 mmHg**
**Acute kidney failure**	• diagnosis code: N17; no such diagnosis within 60 days from the index date<br>• medical records: terms related to acute kidney failure such as 'AKI', 'azotemia', 'toxic nephropathy', 'renal impairment', 'renal failure', 'prerenal failure', 'oliguria', 'anuria'<br>• clinical examination: one or more of the following criteria are satisfied<br>– Increasing of serum creatinine by more than 0.3 mg/dL within 48 hours<br>– * **Increasing of serum creatinine by more than 1.5 times from the baseline**<br>– Reduction of urine volume to less than 0.5 mg/kg/h for 6 hours
**Cardiac failure**	• diagnosis code: I50; no such diagnosis within 4 months from the index date<br>• medical records: terms related to cardiac failure such as 'pulmonary edema', 'hepatic congestion', 'cardiopulmonary failure', 'acute pulmonary edema', 'acute cardiac failure', 'right ventricular failure'<br>• clinical examination: one or more of the following criteria are satisfied<br>– * **BNP > 100ng/L or NT-proBNP > 125 ng/L**

	– Reduction of ejection fraction to less than 50% on echocardiography<br>– Confirmed heart failure by TTE, CT, or angiography
**Ischemic heart disease**	• diagnosis code: I20, I21, I22, I23, I24, I25; no such diagnosis within 4 months from the index date<br>• medical records: terms related to ischemic heart disease such as 'angina pectoris', 'unstable angina', 'myocardial infarction', 'coronary disease', 'coronary artery occlusion', 'acute coronary syndrome', 'coronary artery stenosis', 'cardiomyopathy stress', 'abnormal ECG ST-T seg', abnormal ECG T wave', 'CKMB elevation', 'Troponin I elevation'<br>• [†]**clinical examination: one or more of the following criteria are satisfied**<br>– CKMB > 6.6 ng/mL<br>– Troponin I > 0.028 ng/mL<br>– Abnormal ST segment on ECG<br>– Abnormal T wave on ECG<br>– QTc > 500 ms or increased by more than 60 ms from the baseline on ECG
**Diarrhea**	• diagnosis code: K58.0, K59.1, P78.3, R19.7; no such diagnosis within 60 days from the index date<br>• medical records or nursing records: terms related to diarrhea such as [†] **'4 or more bowel movements per day'**, 'loose stool', 'diarrhea'<br>• drugs: newly administration of antidiarrheal drugs such as smectite and loperamide
**Pyrexia**	• diagnosis code: R509; no such diagnosis within 60 days from the index date<br>• medical records or nursing records: terms related to fever such as 'fever'<br>• clinical examination: [†]**body temperature > 38°C**
**Cough**	• diagnosis code: R05; no such diagnosis within 60 days from the index date<br>• medical records or nursing records: terms related to cough such as [*] **'cough', 'dry cough'**

	• drugs: newly administration of antitussives or expectorants such as ambroxol
**Upper respiratory infection**	• diagnosis code: J00-J06, J39, A36.8, A36.9; no such diagnosis within 14 days from the index date<br>• medical records: terms related to upper respiratory infection such as † **'URI', 'nasopharyngitis', 'pharyngitis', 'sinusitis', 'laryngitis', 'tonsillitis'**<br>• clinical examination: confirmed sinusitis on PNS routine examination
**Hypokalemia**	• diagnosis code: E876; no such diagnosis within 60 days from the index date<br>• medical records: terms related to hypokalemia such as'hypokalemia', 'decreased potassium levels'<br>• drugs: newly administration of potassium chloride<br>• ***clinical examination: K < 3.0 mmol/L**
**Muscle spasm**	• diagnosis code: F45.8, F95.1, F95.9, G11.4, G50.0, G81.1, G82.20, M54.08, M62.40, M62.838, R25.0, R26.0, R29.0, R56.9; no such diagnosis within 60 days from the index date<br>• medical records or nursing records: terms related to muscle spasm such as *† **'muscle spasms', 'cramp'**
**Thrombocytopenia**	• diagnosis code: D691, D694, D695, D696; no such diagnosis within 14 days from the index date<br>• medical records: terms related to thrombocytopenia such as 'thrombocytopenia', 'plt decrease'<br>• procedure: platelet transfusion<br>• clinical examination: ***platelet count < 100 x 10^9/L** or platelet count decreased by more than 30% from the baseline

The diagnostic codes are according to ICD-10. *CTCAE grade 1; †CTCAE grade 2

Supplementary Table S4. Definition of the covariates.

Criteria	Definitions	Collection Date
**Surgery**	• Surgery records in medical records	21 days
**Active infection**	Diagnosis code AND drugs<br>• diagnosis code: A00-99, B00-99<br>• drugs: systemic antibiotics, antivirals, antifungals	14 days
**HIV**	• diagnosis code: B24	14 days
**Active B or C hepatitis**	Diagnosis code AND drugs<br>• diagnosis code: B169, B171, B182, B188<br>• drugs<br>– Type B hepatitis: pegylated interferon alfa 2a, entecavir, tenofovir DF, tenofovir AF, besifovir, lamivudine, telbivudine, clevudine, adefovir<br>– Type C hepatitis: Ledipasvir/sofosbuvir, elbasvir/grazoprevir, daclatasvir+sofosbuvir, daclatasvir +asunaprevir, glecaprevir/pibrentasvir, sofosbuvir /velpatasvir, sofosbuvir+ribavirin, pegylated interferon alpha+ribavirin, elbasvir/grazoprevir+sofosbuvir, sofosbuvir/velpatasvir/voxilaprevir, ombitasvir, paritaprevir/ritonavir (+/- dasabuvir)	21 days
**Other malignancy**	• diagnosis code: C00-C49, D469<br>• medical records: other malignancy	3 years
**Peripheral neuropathy**	• diagnosis code: G629<br>• medical records: peripheral neuropathy<br>• drugs: gabapentin, pregabalin, duloxetine	14 days
**Ongoing graft-vs-host disease**	Diagnosis code AND drugs<br>• diagnosis code: T860<br>• drugs: methylprednisolone, sirolimus (mTOR), ruxolitinib, alemtuzumab, basiliximab, tacrolimus (CNI), cyclosporine, abatacept, etanercept, infliximab, mycophenolate mofetil, toxilizumab,	21 days

	methotrexate, ibrutinib, imatinib, interleukin 2, rituximab	
**Pleural effusions, ascites**	Diagnosis code AND procedures<br>• diagnosis code: J90, R18<br>• procedures: anterior chamber paracentesis, thoracentesis	14 days
**Myocardial infarction**	• diagnosis code: I21<br>• medical records: cardiac infarction, coronary embolism, coronary occlusion, coronary rupture, coronary thrombosis, infarction of heart, myocardium, or ventricle, ST elevation (STEMI) myocardial infarction	4 months
**Heart failure**	• diagnosis code: I50<br>• medical records: left ventricular failure, systolic (congestive) heart failure, diastolic heart failure, combined systolic and diastolic heart failure, right heart failure, high output heart failure, biventricular heart failure, end stage heart failure, ventricular failure, cardiac failure, heart decompensation, myocardiac failure	4 months
**Angina**	• diagnosis code: I200<br>• medical records: angina pectoris, angina at rest, myocardial pre-infarction syndrome, accelerated angina, crescendo angina, de novo effort angina, intermediate coronary syndrome	4 months
**Coronary artery disease**	• diagnosis code: I251, I200<br>• medical records: coronary artery embolism, coronary artery occlusion, coronary artery stenosis, coronary artery thrombosis	4 months
**Ventricular arrhythmia**	• diagnosis code: I470, I49.9, I49.0, I47.2<br>• medical records: ventricular arrhythmia, ventricular fibrillation and flutter, ventricular tachyarrhythmia, ventricular tachycardia	4 months
**Sick sinus syndrome**	• diagnosis code: I495<br>• medical records: bradycardia tachycardia	4 months

	syndrome, sinus node dysfunction, tachycardia-bradycardia	
**Acute ischemia**	• diagnosis code: I24, I240, I248, I249<br>• medical records: acute coronary syndrome	4 months
**Conduction system abnormalities**	• diagnosis code: I44, I45<br>• medical records: cardiac dysrhythmia, conduction disorder of the heart, Adams-Stokes syndrome, Wolff-Parkinson-White syndrome, Brugada syndrome, Frederick's syndrome, Lenegre's disease	4 months
**Hypertension**	• diagnosis code: I10<br>• medical records: SBP≥140 mmHg, DBP≥ 90 mmHg<br>• drugs: ACE inhibitors, angiotensin receptor blocker, beta-blockers, calcium channel blockers, diuretics	4 months
**Diabetes mellitus**	• diagnosis code: E10, E11<br>• medical records: HbA1c>7%, pre-prandial capillary plasma glucose>130mg/dl, peak postprandial capillary plasma glucose of >180mg/dl<br>• drugs: sulfonylureas, metformin, alpha-glucosidase inhibitors, meglitinides, thiazolidinediones, DPP-4 inhibitors, SGLT2 inhibitors, GLP-1 receptor agonists, insulins	14 days

The diagnostic codes are according to ICD-10. The collection date means the screening period before the index date.

Supplementary Table S5. Reasons for chemotherapy discontinuation

Reason	KRd (n = 69)	Rd (n = 69)	*p* Value
Adverse drug reactions	13 (18.8)	15 (21.7)	0.67
Disease progression	12 (17.4)	15 (21.7)	0.52
Death	1 (1.4)	4 (5.8)	0.17
Unknown	3 (4.3)	0 (0.0)	-
Follow up loss	1 (1.4)	2 (2.9)	0.56

no.(%)

Supplementary Table S6. Sensitivity analysis by contemporary comparator

	KRd (n=69)		Rd (n=47)		Adjusted HR¥ (95% CI)
	**Events**	**Events/100-Patients Cycle**	**Events**	**Events/100-Patients Cycle**	
**Non-hematologic adverse events**					
Dyspnea	30	9.84	13	2.95	**2.48 (1.21–5.09)**
Hypertension	23	6.04	13	2.89	**2.34 (1.08–5.06)**
Acute renal failure	11	2.66	6	1.18	2.03 (0.69–5.95)
Cardiac failure	11	2.66	7	1.43	1.83 (0.66–5.09)
Ischemic heart disease	6	1.38	6	1.28	1.33 (0.39–4.54)
Diarrhea ¶	17	4.51	12	2.83	1.08 (0.48–2.42)
Cough ¶	11	2.95	7	1.45	1.66 (0.58–4.79)
Pyrexia ¶	28	8.14	28	8.14	1.96 (0.95–4.04)
Upper respiratory tract infection ¶	11	2.96	6	1.27	1.41 (0.48–4.16)
Hypokalemia ¶	9	2.05	3	0.6	2.44 (0.61–9.83)
Muscle spasm ¶	9	2.24	2	0.38	3.04 (0.59–15.55)
**Hematologic adverse events**					
Thrombocytopenia ¶	38	12.71	19	5.03	**1.98 (1.09–3.59)**

¶ Hematologic and non-hematologic adverse events occurred more than 25% in the ASPIRE study with the difference of more than 5% between the two groups. ¥ HR was adjusted for age. KRd, combination regimen of carfilzomib, lenalidomide and dexamethasone; Rd, combination regimen of lenalidomide and dexamethasone

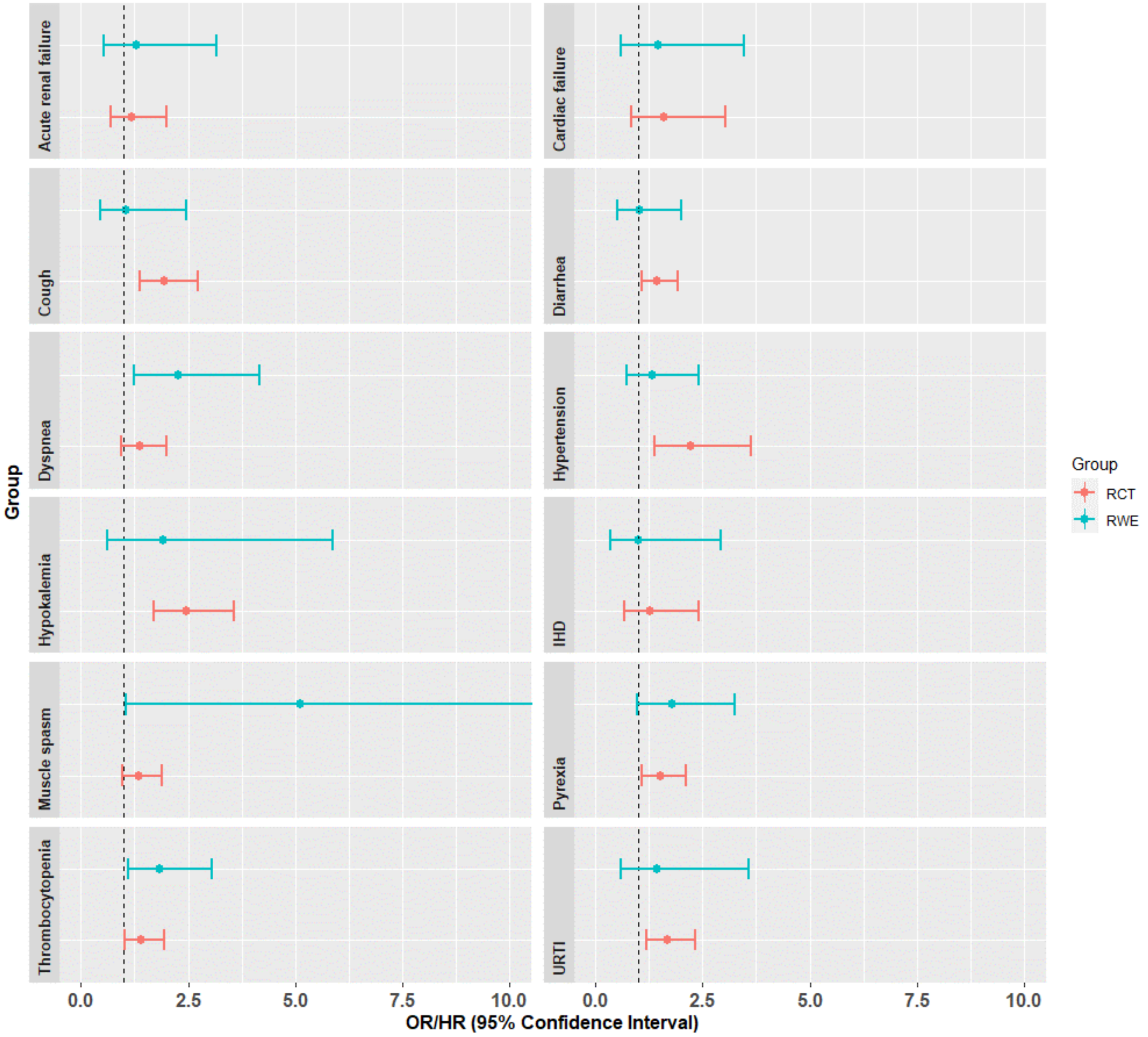

Supplementary Figure S1. Forest plot of HR in RWE and OR in ASPIRE trial for each adverse reaction. IHD, Ischemic heart disease; RCT, randomized clinical trials; RWE, real world evidence; URTI, Upper respiratory tract infection
